# Modeling the novel SERD elacestrant in cultured fulvestrant-refractory HR-positive breast circulating tumor cells

**DOI:** 10.1007/s10549-023-06998-w

**Published:** 2023-06-15

**Authors:** Taronish D. Dubash, Aditya Bardia, Brian Chirn, Brittany A. Reeves, Joseph A. LiCausi, Risa Burr, Ben S. Wittner, Sumit Rai, Hitisha Patel, Teeru Bihani, Heike Arlt, Francois-Clement Bidard, Virginia G. Kaklamani, Philippe Aftimos, Javier Cortés, Simona Scartoni, Alessio Fiascarelli, Monica Binaschi, Nassir Habboubi, A. John Iafrate, Mehmet Toner, Daniel A. Haber, Shyamala Maheswaran

**Affiliations:** 1 Massachusetts General Hospital Cancer Center and Harvard Medical School, Boston, MA 02114 USA; 2 Radius Health, Inc, Waltham, MA 02451 USA; 3 Institut Curie, Paris, Saint Cloud France; 4 University of Texas Health Sciences Center, Houston, TX 77030 USA; 5 Institut Jules Bordet—Université Libre de Bruxelles, Brussels, Belgium; 6 International Breast Cancer Center (IBCC), Quiron Group, Barcelona, Spain; 7 Menarini Group, Pomezia, Italy; 8 Stemline Therapeutics/Menarini Group, New York, NY 10022 USA; 9 Center for Engineering in Medicine, Department of Surgery, Massachusetts General Hospital and Harvard Medical School, Boston, MA 02114 USA; 10 Howard Hughes Medical Institute, Bethesda, MD 20810 USA

**Keywords:** ER + metastatic breast cancer, Selective estrogen receptor degrader (SERD), Fulvestrant resistance, Circulating tumor cells (CTCs), Elacestrant

## Abstract

**Purpose:**

Metastatic hormone receptor-positive (HR+) breast cancer initially responds to serial courses of endocrine therapy, but ultimately becomes refractory. Elacestrant, a new generation FDA-approved oral selective estrogen receptor degrader (SERD) and antagonist, has demonstrated efficacy in a subset of women with advanced HR+breast cancer, but there are few patient-derived models to characterize its effect in advanced cancers with diverse treatment histories and acquired mutations.

**Methods:**

We analyzed clinical outcomes with elacestrant, compared with endocrine therapy, among women who had previously been treated with a fulvestrant-containing regimen from the recent phase 3 EMERALD Study. We further modeled sensitivity to elacestrant, compared with the currently approved SERD, fulvestrant in patient-derived xenograft (PDX) models and cultured circulating tumor cells (CTCs).

**Results:**

Analysis of the subset of breast cancer patients enrolled in the EMERALD study who had previously received a fulvestrant-containing regimen indicates that they had better progression-free survival with elacestrant than with standard-of-care endocrine therapy, a finding that was independent estrogen receptor *(ESR1)* gene mutations. We modeled elacestrant responsiveness using patient-derived xenograft (PDX) models and in ex vivo cultured CTCs derived from patients with HR+breast cancer extensively treated with multiple endocrine therapies, including fulvestrant. Both CTCs and PDX models are refractory to fulvestrant but sensitive to elacestrant, independent of mutations in *ESR1* and Phosphatidylinositol-4,5-Bisphosphate 3-Kinase Catalytic Subunit Alpha (*PIK3CA)* genes.

**Conclusion:**

Elacestrant retains efficacy in breast cancer cells that have acquired resistance to currently available ER targeting therapies. Elacestrant may be an option for patients with HR+/HER2- breast cancer whose disease progressed on fulvestrant in the metastatic setting.

Translational Relevance.

Serial endocrine therapy is the mainstay of management for metastatic HR+breast cancer, but acquisition of drug resistance highlights the need for better therapies. Elacestrant is a recently FDA-approved novel oral selective estrogen receptor degrader (SERD), with demonstrated efficacy in the EMERALD phase 3 clinical trial of refractory HR+breast cancer. Subgroup analysis of the EMERALD clinical trial identifies clinical benefit with elacestrant in patients who had received prior fulvestrant independent of the mutational status of the *ESR1* gene, supporting its potential utility in treating refractory HR+breast cancer. Here, we use pre-clinical models, including ex vivo cultures of circulating tumor cells and patient-derived xenografts, to demonstrate the efficacy of elacestrant in breast cancer cells with acquired resistance to fulvestrant.

**Supplementary Information:**

The online version contains supplementary material available at 10.1007/s10549-023-06998-w.

## Introduction

Metastatic Hormone Receptor-positive (HR+) breast cancer remains the predominant cause of breast cancer mortality in women, despite advances in targeted therapies [1]. Breast cancers expressing Estrogen Receptor alpha (ERα, encoded by the *ESR1* gene) are dependent on estrogen-induced signaling for proliferation, and they are highly susceptible to an array of hormonal interventions targeting this pathway. These include treatment with selective estrogen receptor modulators (SERMS) which compete for estrogen binding, aromatase inhibitors (AI) that suppress estrogen biosynthesis, and selective estrogen receptor degraders (SERD) which are thought to enhance degradation of the receptor [1–4]. The sequential use of these drugs, often in combination with CDK4/6 and PI3K inhibitors, has resulted in dramatically prolonged survival of women with metastatic breast cancer [5–10]. However, breast cancer cells ultimately evolve drug resistance mechanisms, many of which are complex and overlapping in their drug specificity profiles [11–13]. Among these, mutations in *ESR1* itself have merged as an important driver of resistance to hormonal agents [14–18]. There is thus a major unmet need for new classes of drugs that target ER in breast cancer cells that have preserved their dependence on this signaling pathway but have become refractory to currently available agents.

Fulvestrant (Faslodex), given intramuscularly (IM), is currently the only FDA-approved SERD for the treatment of metastatic HR+ breast cancer. However, despite its *in vitro* potency, fulvestrant has poor pharmacokinetic properties and it has relatively modest clinical efficacy as a single agent [19]. Multiple candidate oral SERDs, with improved bioavailability and enhanced ER degradation capacity, have recently entered clinical trials, including elacestrant, giredestrant, camizestrant, and imlunestrant, among others [20, 21].

Elacestrant is a novel FDA-approved small molecule drug which displays antitumor activity both as a single agent, as well as in combination with CDK4/6 inhibitors in breast cancer cell lines and xenografts [22, 23]. Initial clinical trials demonstrated activity in women with advanced metastatic HR+ breast cancer [24], including patients whose tumors had acquired *ESR1* mutations, leading to the Phase 3 randomized EMERALD clinical trial in 2nd–3rd line setting*,* reporting a statistically significant improvement in Progression Free Survival (PFS) [25]. Of note, some patients in the study had rapid disease progression as reflected by initial drop in PFS curves, irrespective of treatment with elacestrant or standard-of-care regimens; however, a major difference in PFS emerged subsequently, with a 12 month PFS of 22.3% for elacestrant, compared with 9.4% for standard-of-care regimens. This biphasic response raises the possibility that advanced HR+ cancer is heterogeneous with respect to ER signaling, and that only the subset of cases with preserved dependence on that pathway are susceptible to elacestrant. The presence of *ESR1* mutations acquired during prior endocrine therapies was correlated with improved efficacy for elacestrant (12 month PFS: 26.8% vs 8.2%) [25], consistent with *ESR1* mutations being a surrogate marker for cancers with preserved dependence on ER signaling.

CTCs are shed into the blood by metastatic breast cancers, where they constitute potential precursors of further metastatic dissemination [26]. While only a subset of CTCs may survive the stressful environment in the bloodstream giving rise to new metastatic lesions, viable cells isolated from blood samples can be expanded *ex vivo*, where they provide a unique resource to monitor and functionally analyze breast cancer cells that have been exposed to multiple therapeutic regimens during the course of therapy [16]. We have previously shown that cultured breast CTCs faithfully recapitulate both original and acquired mutations identified in patient tumors and that their response to targeted therapies can help distinguish acquired driver mutations with therapeutic impact from passenger mutations [12, 16]. CTCs cultured from patients with breast cancer and with melanoma also exhibit a high degree of heterogeneity and cellular plasticity, reflected in distinct subpopulations with different degrees of sensitivity to therapeutic agents [12, 16, 27, 28]. As such, clinically and genetically annotated breast CTC cell lines, established *in vitro* from patients who were heavily treated with diverse endocrine regimens, may provide insight into the efficacy of elacestrant in refractory HR+ breast cancer.

In this translational study, we tested the efficacy of elacestrant on *ex vivo* CTC cultures, capturing the diversity and treatment histories of multiple patients with refractory breast cancer and patient-derived mouse xenograft models with serial exposures to fulvestrant. In addition, we conducted reanalysis of the EMERALD randomized clinical trial to evaluate efficacy of elacestrant vs standard endocrine therapy among patients who had received prior fulvestrant.

## Material and methods

### Clinical trial (EMERALD) analysis

The study design and primary results from the multicenter, randomized, open-label, phase 3 clinical study (EMERALD; ClinicalTrials.gov, NCT03778931) have been published previously [25, 29]. Briefly, the clinical trial included postmenopausal women or men 18 years or older with histologically or cytologically proven ER-positive/HER2-negative breast adenocarcinoma, and either locoregionally recurrent or metastatic disease, Eastern Cooperative Oncology Group performance status 0 or 1, prior treatment with one or two prior lines of endocrine therapy for advanced/metastatic disease including previous CDK4/6 inhibitor treatment in combination with fulvestrant or an AI, and evaluable/measurable disease per Response Evaluation Criteria in Solid Tumors (RECIST) v1.1. Patients were randomized 1:1 to elacestrant or standard-of-care endocrine therapy (either fulvestrant or aromatase inhibitor). Standard-of-care treatment was per investigator’s choice based on protocol guidance, which recommended use of a different endocrine therapy than the patient had received previously. *ESR1* mutational status at baseline was evaluated in cell-free circulating DNA using the Guardant360^®^ test (Guardant Health, Redwood City, CA), and tumor assessments were conducted with computed tomography/magnetic resonance imaging (CT/MRI) at baseline and every 8 weeks. Two primary endpoints of the trial were progression-free survival (PFS) in all patients and in patients with detectable *ESR1* mutation, each assessed by blinded independent central review (BICR) utilizing standard RECIST v1.1 criteria, and the results have been published previously [29]. The trial met regulatory requirements and was performed in accordance with ethical principles consistent with the Declaration of Helsinki and International Council of Harmonisation/Good Clinical Practice. For this project, we evaluated progression-free survival stratified by prior use of fulvestrant, a pre-defined secondary endpoint in the EMERALD trial. Progression-free analyses were performed using standard Kaplan–Meier methods based on the intention-to-treat populations for all patients and patients with *ESR1* mutation. Hazard ratio and 95% confidence interval (CI) for the difference between treatment groups were estimated using the stratified Cox regression model, including treatment as a variable, and analyzed using the stratified log-rank test, similar to primary analysis.

**Fig. 1:**
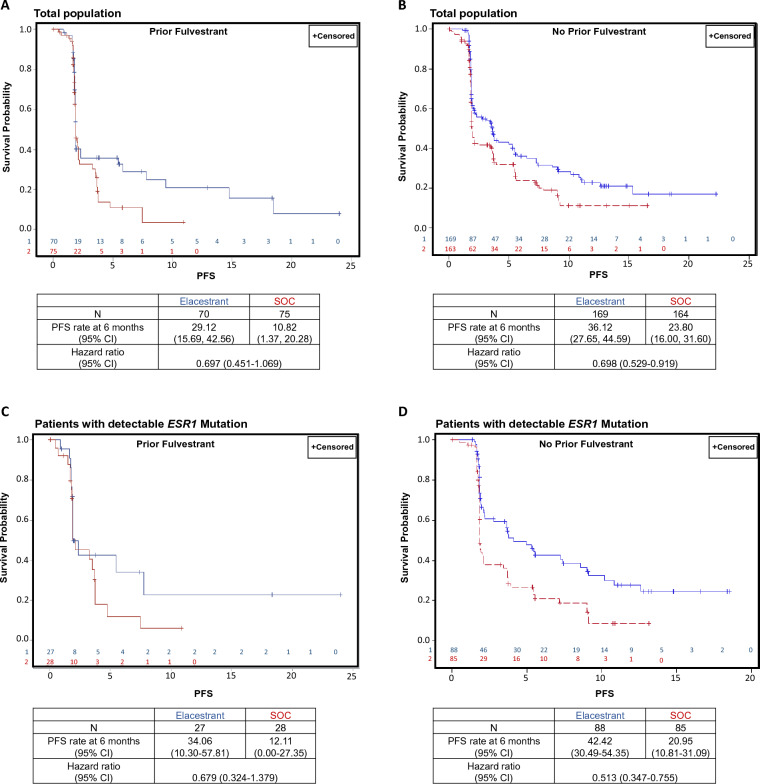
Clinical outcomes of patients within the EMERALD trial of elacestrant vs standard endocrine therapy, stratified by prior fulvestrant treatment. **A** and **B**: Kaplan–Meier estimates of progression-free survival (PFS) assessed by blinded independent central review are shown for elacestrant versus standard of care (SOC) in patients with prior treatment with fulvestrant (**A**) and in patients without prior treatment with fulvestrant (**B**). All cases derived from the full study population. (**C** and **D**): Kaplan–Meier estimates of PFS for elacestrant versus SOC in patients with prior treatment with fulvestrant (**C**) or without prior treatment with fulvestrant (**D**), among patients with detectable *ESR1* mutations. Interaction P value was independent of fulvestrant treatment

### Measuring elacestrant responses in a PDX mouse model

**Fig. 2:**
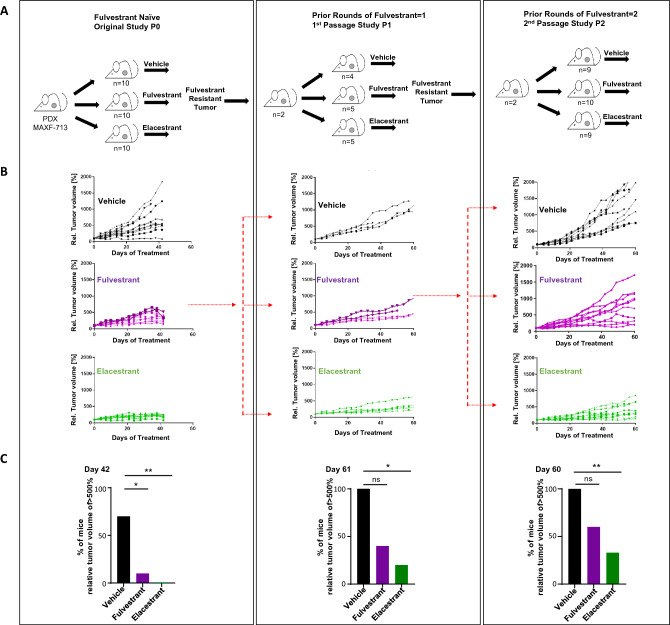
Fulvestrant-refractory breast cancer is sensitive elacestrant in a PDX model **A** Schematic representation of elacestrant and fulvestrant treatment of PDX breast tumor model, MAXF-713 derived from untreated ER+/PR+/Her2- breast cancer patient (P0) and subsequent treatment rounds of the fulvestrant-refractory tumors (P1, P2) with either elacestrant and fulvestrant. **B** Curves showing the relative tumor volumes in mice treated with vehicle, fulvestrant and elacestrant over time, for successive passages (P0, P1 and P2). The tumors represented in the darker shaded curves were predecessors for the subsequent passage. **C** Bar graphs showing the percentage of mice with relative tumor volume greater than 500% for each of the treatment cohorts. (P0: vehicle control vs fulvestrant *p* = 0.0197, vehicle control vs elacestrant *p* = 0.00309; P1: vehicle control vs fulvestrant *p* = 0.16, vehicle control vs elacestrant *p* = 0.0476; P2: vehicle control vs fulvestrant *p* = 0.086, vehicle control vs elacestrant *p* = 0.009)

All study protocols were reviewed by Radius, approved by Institutional Animal Care and Use Committees (IACUC), and conducted in accordance with US and International regulations for protection of laboratory animals. MAXF-713 patient-derived xenograft fragments (*ESR1 PIK3CA* Wildtype) were implanted into intact athymic nude mice, and 17ß-estradiol supplied in the drinking water [22]. All mice were housed in pathogen-free housing in individually ventilated cages with sterilized and dust-free bedding cobs, access to sterilized food and water ad libitum, under a 14h light/10h dark artificial light cycle and controlled room temperature and humidity. Tumors were measured twice/week with vernier calipers; volumes were calculated using the formula: (L × W^2^) × 0.5. Elacestrant was administered orally, once daily for the duration of the study. In the first study, 60 mg/kg elacestrant was administered once daily; in subsequent studies, 30 mg/kg elacestrant was administered once daily; fulvestrant was administered once/week subcutaneously (s.c). To develop a fulvestrant-resistant PDX model, mice were treated with a clinically relevant dose of fulvestrant (3 mg/dose/week) [22]. Tumors growing in the presence of fulvestrant were allowed to grow to >1500 mm^3^ and then harvested and retransplanted into a new cohort of mice; this was considered to be passage (P1). This process was repeated to establish a fulvestrant-resistant *in vivo* model (Fig. 2A). Percent relative tumor volume (Rel. tumor volume (%)) was calculated using the following formula: % rel. TV = TV(day x)/TV(day 0)*100. TV = tumor volume, *t* = treatment, *c* = control, avg = average. *p*-values were computed by two-sided Fisher’s exact test.

### In vivo study using breast cancer PDX model (Champions Oncology)

Athymic Nude-Foxn1nu mice were bilaterally implanted with fragments from Champions TumorGraft^®^ model CTG-1260, representing human breast cancer. CTG-1260 was derived from a patient who was treated with fulvestrant for 9 months; the patient initially responded and eventually relapsed. The biopsy (from which the CTG-1260 PDX model was derived) was performed during the relapse on fulvestrant treatment. After the tumors reached 1000–1500 mm^3^, they were harvested, and the tumor fragments were implanted s.c. in the left flank of the female study mice. Each animal was implanted with a specific passage lot and documented. Tumor growth was monitored twice a week using digital calipers, and the tumor volume (TV) was calculated using the formula (0.52 × [length × width^2^]). When the TV reached 150–300 mm^3^, animals were matched by tumor size and assigned into control or treatment groups (*n* = 10/group), and dosing was initiated on Day 0. Tumor size and body weight were measured twice weekly, and the study was terminated and completed on Day 55. In the PDX model, CTG-1260 elacestrant was administered orally at 30 mg/Kg Q1×56 d. All experimental procedures were performed according to the guidelines of the Institutional Animal Care and Use Committee (IACUC) of Champions Oncology. Elacestrant dosing solution of 3 mg/mL was prepared by adding 0.5% Methylcellulose in sterile water to elacestrant powder, stirred for 4 min, and sonicated to form a transparent solution.

### Enrichment of Patient-derived CTCs

For CTC collection, written informed consent was obtained from patients with metastatic breast cancer (MBC) as per institutional review board (IRB)-approved protocol (DF/HCC 05-300). CTCs were isolated using the microfluidic CTC-iChip as previously described [16, 28]. In brief, whole blood was incubated with biotinylated antibodies against CD45 (clone 2D1; R&D Systems), CD66b (clone 80H3; AbD Serotec), and CD16 (Janssen Diagnostics) followed by incubation with Dynabeads MyOne Streptavidin T1 (Invitrogen) to achieve magnetic labeling of leukocytes. Blood specimens were then processed through the CTC-iChip to deplete leukocytes, while leaving CTCs untagged and unmanipulated. The leukocyte-depleted product was collected in CTC culture media for the derivation of patient-specific CTC cultures [16].

### Ex vivo CTC cultures

CTC cultures were grown at 37 ^0^C under hypoxic conditions (4% O2) in ultra-low attachment flasks (Corning) in CTC media comprising of RPMI-1640, 1X antibiotic/antimycotic supplemented with bFGF (20 ng/ml), EGF (20 ng/ml), and 1X B27 (Life Technologies). Cultures were routinely checked for mycoplasma with the MycoAlert, Lonza Kit. Cell cultures and patient blood were tested for authentication via STR profiling by Genetica DNA Laboratories (a LabCorp brand; Burlington, NC) using the commercially available PowerPlex^®^ 16HS amplification kit (Promega Corporation; mouse marker included) and GeneMapper ID v3.2.1 software (Applied Biosystems). Brx50 and Brx68 have been described previously by Yu et al [16]. Brx211, Brx250, and Brx394 have been previously described by Brett et al [30]. Mutational screening for *98* known cancer genes was performed using the MGH SNaPshot-NGS clinical assay as described elsewhere [31]. The assay includes single-nucleotide variants (SNVs) within ESR1 as well as ESR gene fusions [32].

### Immunoblotting

Proteins were isolated from 2 to 5×10^5^ cells using RIPA buffer (BP-115X) supplemented with the HaltTM Protease inhibitor cocktail (Thermo Scientific 78425). Protein lysates (10μg each) were separated on SDS/4-15% polyacrylamide gels (Bio-Rad) and transferred to nitrocellulose membranes (Invitrogen). The blots were incubated with antibodies directed against GAPDH (sc-47724, mouse monoclonal, 1:1000), Estrogen Receptor α (D8H8) (Cell signaling-8644, Rabbit monoclonal, 1:500) and with the relevant secondary antibodies which were HRP conjugated (Anti-rabbit IgG, HRP-linked Antibody cell signaling-7074, Anti-mouse IgG, HRP-linked Antibody Cell signaling-7076) and visualized using Clarity Western ECL Substrate (BIORAD) and G box (Syngene).

### Immunohistochemistry (IHC) staining

Staining was performed at MGH core facility using automated staining platform (Ventana Discovery Ultra). CTC cultures were formalin-fixed before embedding into blocks with paraffin. 10 mM-thick sections were cut and stained for Estrogen Receptor (1:500 ab16660) using sodium citrate antigen retrieval method. Images were acquired using the Aperio Scanscope slide scanner (Leica Biosystems).

### Drug sensitivity testing

Breast CTC cell lines (1,000 cells per well) were seeded in 96-well ultra-low attachment plates (Corning). Increasing concentrations of elacestrant (Radius Health) and fulvestrant (Selleckchem S1191) were added to quadruple samples. The cells were incubated with the drugs for 7 days under hypoxic conditions. Viability was measured using CellTiter-Glo Luminescent Cell Viability Assay per manufacturer’s instructions.

### Quantitative Real time PCR

Total RNA was extracted using the RNeasy Mini Kit (Qiagen). RNA (1µg) was used to generate cDNA with the superscript III First Strand synthesis system (Life Technologies). Reactions were amplified and analyzed in triplicate using the ABI 7500 Real-Time PCR System.

Primer Sequence (5’->3’)

ESR1-alpha

Forward Primer: GCCTTCTTCAAGAGAAGTATT

Reverse Primer: TTTCGTATCCCACCTTTCATC,

GREB1

Forward Primer: CCATCGGCTTTAGGTATCTTG

Reverse Primer: GCTCTCATACTTAGCTCTGTTC,

PGR

Forward Primer: CTGTCATTATGGTGTCCTTACC

Reverse Primer: AGTCATTTCTTCCAGCACATAA,

GAPDH

Forward Primer: TGTAGTTGAGGTCAATGAAGGG

Reverse Primer: ACATCGCTCAGACACCATG

### Capillary electrophoresis immunodetection

Cell lysates were prepared using the radioimmunoprecipitation (RIPA) buffer (Thermo Fisher Scientific 89901) supplemented with Complete Mini protease inhibitors (Roche) and PhosSTOP phosphatase inhibitors (Roche). Proteins of interest were analyzed by JESS (Protein Simple Inc.) capillary electrophoresis-based immunodetection system. Reagents and equipment were purchased from Protein Simple and samples were analyzed following the manufacturer’s instructions. All experiments were performed in triplicate. Anti-ER antibody (CST #8644) anti-GAPDH (SCBT 47724) were multiplexed and detected with CHEMI and NIR channel, respectively.

## Results

### Clinical outcomes with elacestrant in fulvestrant-treated patients within the EMERALD Study

The study population in the recently completed the phase 3 EMERALD Study [29] includes patients with HR+ metastatic breast cancer who had disease progression on at least one prior endocrine therapy, including combination with CDK 4/6 inhibitors, reflecting contemporary clinical practice. We analyzed data from the EMERALD trial, to determine clinical responses to elacestrant, compared with standard third-line endocrine therapy, among women who had previously been treated with a fulvestrant-containing regimen. Of the 446 enrolled patients, 145 (32.5%) had received fulvestrant as part of prior endocrine therapy, either in first- or second-line settings. As expected, genomic profiles of tumors and clinical treatment histories at the time of treatment with elacestrant were diverse, consistent with contemporary clinical care of advanced breast cancer [29]. Consistent with the overall results of the EMERALD trial [29], a subset of patients experienced rapid disease progression (<2 months) following treatment with either elacestrant or standard endocrine therapy, leading to the suggestion that their cancers were no longer biologically dependent on ER signaling. Among patients who did not have rapid progression and presumably retained endocrine sensitive disease, those who received elacestrant had better PFS compared to those with standard endocrine therapy, irrespective of past treatment with fulvestrant: *fulvestrant-treated,* 6 months PFS = 29.12% on elacestrant (n-70) vs 10.82% on standard endocrine therapy (n=75), HR: 0.697; f*ulvestrant-naïve:* 6 months PFS = 36.12% on elacestrant (n=169) vs 23.80% on standard endocrine therapy (n=164), HR = 0.698. (Fig. 1A, B). Similarly, among patients with detectable *ESR1*-mutant breast cancer, patients treated with elacestrant had better PFS compared to those receiving standard endocrine therapy, irrespective of past fulvestrant treatment: *fulvestrant-treated*, 6 months PFS = 34.06% on elacestrant (*n*=27) vs 12.11% on standard endocrine therapy (*n*=28), HR: 0.679; *fulvestrant-naïve:* PFS rate at 6 months= 42.42% on elacestrant (*n*=88) vs 20.95% on standard endocrine therapy (n=85), HR = 0.513; (Fig. 1C, D). Thus, prior treatment and disease progression on fulvestrant does not reduce the subsequent response to elacestrant therapy (p-value for fulvestrant interaction =0.97).

Of note, the hazard ratio we previously reported was in the subgroup analysis (Forest plot) [29]. Accordingly, the results were unstratified as per routine subgroup analysis. However, in the current analysis, we did a deeper evaluation and specifically calculated the results for the prior fulvestrant subgroup based on Cox survival model with stratification factors, similar to the ones utilized for primary analysis. Accordingly, the sample size is the same but hazard ratios are different.

### Elacestrant inhibits fulvestrant-refractory breast cancer growth in two PDX models

To model in parallel elacestrant and fulvestrant treatment of a PDX breast tumor model, we made use of fulvestrant naive MAXF-713 cells, derived from a 60-year-old patient with untreated ER+/PR+/Her2- breast cancer [22]. MAXF-713 cells are negative for both *ESR1* and *PIK3CA* mutations. Tumor fragments of equal volume were inoculated into athymic nude female mice (P0) and once they reached 100 mm^3^, they were treated with vehicle control, fulvestrant (3 mg/mouse subcutaneous, weekly) or elacestrant (60 mg/kg oral gavage, daily) (*n*=10 mice per group) (Fig. 2). The drug concentrations chosen were based on pharmacokinetic analysis previously described [22]. Briefly, dosing a mouse with 3 mg of elacestrant achieves 1.8× of the plasma concentrations seen in breast cancer patients receiving 500 mg of elacestrant in the clinic. This dose of elacestrant was chosen to achieve clinically relevant concentrations in the mouse PDX model.

Compared with untreated tumors, fulvestrant and elacestrant treatment significantly inhibited tumor growth in mice (Fig. 2B, C). At 8 weeks, two tumors that were proliferating in two individual mice, despite fulvestrant treatment, were viably resected. They were each inoculated into two individual mice to generate enough tumor tissue to inoculate a sufficient number of mice required for the multiple arms of the drug treatment study. (Fig 2A, schema). To avoid the confounding effects of using two different tumors, fragments from both tumors (P1) were equally distributed among the 14 mice to be used in the various arms of the experiment. The same was done to derive the P2 cohort of mice. Once initial tumors were initiated (100mm^3^), P1 mice were randomized for the second round of treatment with vehicle (*n*=4), fulvestrant (*n*=5) or elacestrant (*n*=5). Similarly, after another 8 weeks of treatment, two P1 fulvestrant-resistant tumors were again expanded as described above into 28 mice (P2) for the third cycle of treatment (vehicle: *n*=9, fulvestrant: *n*=10, elacestrant: n=9) For P1 and P2 studies, the dose of elacestrant used was reduced to 30mg/kg. In these serial tumor transplant studies using the MAXF-713 PDX model, fulvestrant-refractory tumors continued to be significantly more sensitive to elacestrant, at the clinically relevant dose of 30mg/kg, compared with fulvestrant (Fig. 2B, C).

**Fig. 3:**
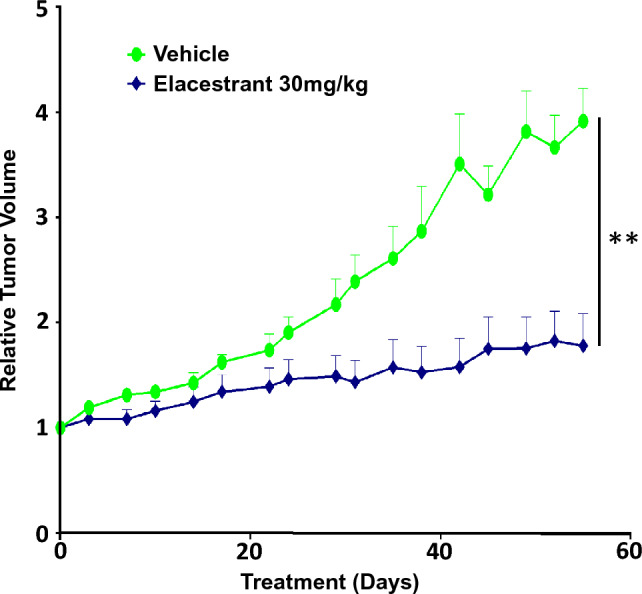
Elacestrant suppresses tumorigenesis in *ESR1* and *PIK3CA* double-mutant breast cancer PDX. The graph shows elacestrant response in the endocrine and palbociclib-resistant ER + /HER2- PDX model of breast cancer (CTG-1260), which harbors the D538G mutation in *ESR1* and two mutations in *PIK3CA*, D350H, and H1047R. Patient-derived cells were injected subcutaneously into athymic Nude-Foxn1nu mice (*n* = 10 ice for each treatment arm)*.* Elacestrant was administered daily at 30 mg/kg. **represents *p*-value < 0.005 using the two-tailed Mann–Whitney rank test

To extend our analysis to breast cancer PDX models harboring mutations that are commonly acquired during course of therapy, we tested the activity of elacestrant in an endocrine and palbociclib-resistant model of ER+/HER2- breast cancer (CTG-1260), which has two mutations in *PIK3CA,* D350H, and H1047R, and the D538G mutation in *ESR1.* CTG-1260 was derived from a patient on fulvestrant for 9 months and initially responded. The CTG-1260 PDX model is derived from the biopsy tissue taken during relapse. Compared with the vehicle-treated controls, elacestrant at a clinically relevant dose (30 mg/kg, daily), demonstrated significant antitumor activity. The mice did not exhibit any weight loss or adverse effects from treatment suggesting good drug tolerability (Fig. 3).

### Ex vivo CTC cultures from hormone-refractory metastatic breast cancer patients are sensitive to elacestrant, independent of ESR1 status

**Fig. 4:**
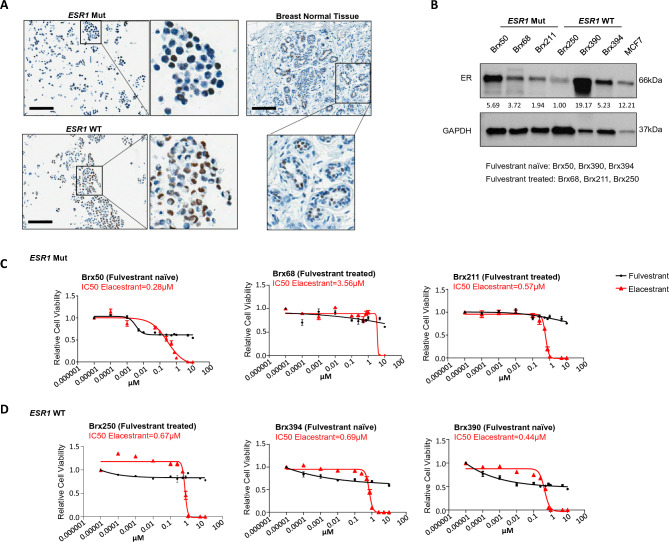
Sensitivity of cultured CTCs from refractory HR+breast cancer patients to elacestrant compared with fulvestrant. **A** Representative images showing nuclear staining for ER in ex vivo CTC cultures harboring either wild-type *ESR1* or mutant *ESR1*. Human normal breast tissue was used as a positive control. High magnification images of the highlighted areas are provided. Scale bar represents 100 µm. **B** Western blot showing ER protein expression in CTC lines with wild-type or mutant *ESR1*. MCF7 was used as a positive control for ER expression. GAPDH is shown as loading control. **C** and **D** Relative cell viability of CTCs treated with fulvestrant (black) or elacestrant (red) for 7 days. CTC cultures were established from hormone-refractory metastatic breast cancer patients, with drug treatment shown for CTC lines with mutant *ESR1*: Brx50, Brx68, Brx211 (**C**) or wild-type *ESR1:* Brx250, Brx394, Brx390 (**D**). IC50 values are shown for elacestrant; fulvestrant was ineffective in achieving complete growth inhibition across all CTC lines

To extend the mouse models to patient-derived specimens from women with advanced HR+ breast cancer, we tested *ex vivo* cultures of CTCs obtained from blood specimens during therapy. We established long-term cultures of CTCs from six patients with refractory, metastatic HR+ breast cancer, using microfluidic depletion of leukocytes fro–20 ml of whole blood, followed by *in vitro* expansion under anchorage independent, hypoxic culture conditions [16, 33]. The patients had received on average eight different treatments following diagnosis of metastatic disease: all six patients had received a SERM; three had received a course of fulvestrant therapy, three a PI3K inhibitor, and three a CDK4/6 inhibitor (Full patient characteristics and treatment history are shown in Table 1 and Sup. Fig. 1. Mutational signatures of CTCs are consistent with those expected for advanced HR+ breast cancer, including the presence of *ESR1* mutations in three of the six lines (Brx50, Brx68, Brx211), and *PIK3CA* mutations in three of the six lines (Brx68, Brx211, Brx394) (Table 1 and Sup. Tables 1 and 2). CTC lines express variable amounts of ER protein, of normal size, consistent with the absence of *ESR1* fusions (Fig. 4A, B). Treatment of all six CTC lines with fulvestrant shows a high degree of resistance, without a measurable IC_50_ (>100 μM); three CTC lines (Brx50, Brx390, BRx394) harbor approximately 45-55% cells resistant to the highest dose of fulvestrant (1 µM). In marked contrast, elacestrant mediates effective cell killing of cultured CTCs, established from patients who received fulvestrant, with a median IC_50_ of 0.62µM (range 0.28–3.56µM), a concentration clinically achievable. (Fig. 4C, D). The three CTC lines harboring *ESR1* mutations (Fig. 4C) and the three lines with wild-type *ESR1* (Fig. 4D) show comparable sensitivity to elacestrant (p=0.45).

**Table 1:** Clinical characteristics of patients from whom CTC cultures were established

Patient ID	Age at Primary Diagnosis (Years)	ER Status (at Primary Diagnosis)	PR Status (at Primary Diagnosis)	HER2 by IHC (at Primary Diagnosis)		Therapy Prior to CTC Collection				ESR1 Mutation	PIK3CA Mutation
						Endocrine		CDK4/6	Chemo		
					AI	Fulvestrant	Others				
Brx50	32	Positive	Positive	0	Yes	No	Tamoxifen	No	Yes	L536P	Absent
Brx68	54	Positive	Negative	2 +	Yes	Yes	No	No	Yes	Y537S	H1047R
Brx211	44	Positive	Not done	Not done	Yes	Yes	SERD Trial	No	Yes	D538G	E110del
Brx250	59	Positive	Positive	0	No	Yes	No	No	Yes	Absent	Absent
Brx390	52	Positive	Positive	1 +	No	No	Tamoxifen	No	No	Absent	Absent
Brx394	76	Positive	Positive	0	Yes	No	No	Yes	Yes	Absent	E542K

### Elacestrant sensitivity in cultured CTCs is unaffected by previous exposure to fulvestrant

**Fig. 5:**
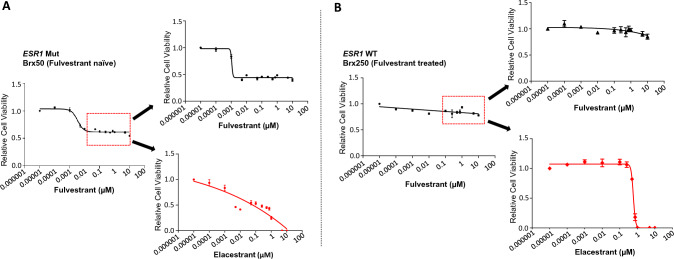
Fulvestrant-resistant CTCs are sensitive to elacestrant and (**B**) The *ESR1*-mutant CTC line, Brx50 (**A**), and the *ESR1* wild-type CTC line, Brx250 were treated with increasing concentrations of fulvestrant for 7 days. The fulvestrant-resistant subpopulation (100 nM; 7 days; red box) was retreated with increasing doses of fulvestrant (black) or elacestrant (red) for 7 days. The graphs show the relative cell viability following treatment with either drug

Of the six CTC lines analyzed, three were derived from patients previously treated with fulvestrant (Brx68, Brx211, Brx250). These lines were not significantly more resistant to elacestrant, compared with CTC lines derived from patients without fulvestrant exposure (Brx50, Brx390, Brx394) (p=0.32). To directly test the effect of fulvestrant exposure on sensitivity to either retreatment with fulvestrant or exposure to elacestrant, we treated CTC lines with fulvestrant for 7 days and then re-tested their drug sensitivity. Across different CTC lines, initial treatment with fulvestrant (100nM) killed from 13.5% to 43.4% of cells with the remaining cells demonstrating slow persistent proliferation (Fig. 5, Sup. Figs. 2, 3). After 7 days of the initial fulvestrant treatment, replating the remaining resistant cells and retreatment with increasing concentrations of fulvestrant showed a drug sensitivity pattern identical to that of untreated cells. Consistent with previous studies, these observations suggest a potentially transient and reversible fulvestrant resistance mechanism [34]. In marked contrast, in the five CTC lines tested, the fulvestrant-resistant cell population is eradicated upon treatment with elacestrant, with IC_50_ values comparable to those of the untreated parental cells (Fig. 5, Sup. Figs. 2, 3).

**Fig. 6:**
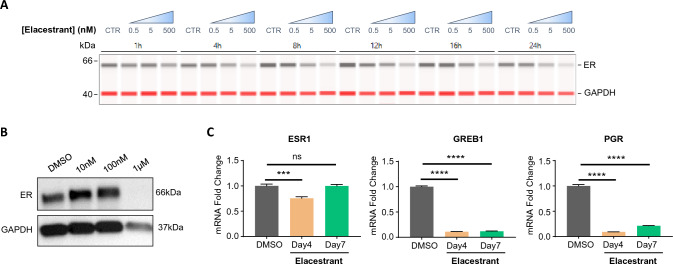
Elacestrant suppresses ER protein and ER-targets in hormone-refractory metastatic CTCs cultured ex vivo **A** MCF7 cells were treated with increasing doses of elacestrant (DMSO, 0.5, 5 and 500 nM) for various times and analyzed by immunocapillary electrophoresis. Western blot shows suppression of ER protein expression by elacestrant, in a time and dose-dependent manner. GAPDH expression is shown as control. **B** Western blot showing reduction in ER protein expression in the CTC line Brx50 (*ESR1*-mutant) with increasing concentrations of elacestrant (5 days). GAPDH, loading control. **C** qRT PCR quantitation of transcripts encoding ER target genes (*GREB1, PGR*) in Brx50 CTCs treated with 100 nM elacestrant (4 and 7 days). Day 4: *ESR1*: *p* = 9.0e-4, *GREB1*: *p* = 9.3e-8, *PGR*: *p* = 1.0e-6); Day 7: *ESR1*: *p* = 0.985, *GREB1*: *p* = 1.2e-7, *PGR*: *p* = 6.3e–7)

Elacestrant has been previously shown to be an ER degrader [22]. Indeed, it suppresses ER protein levels in MCF7 cells, in a time and dose-dependent manner without change in mRNA levels, with degradation evident within 24 hrs at 5nM (Fig. 6A; Sup Fig 4). In cultured CTCs, however, the concentration of elacestrant required to fully suppress ER expression (1 µM) is 10-fold higher than that effective in inhibiting expression of ER target genes, such as *GREB1* and *PGR* (100nM) (Fig. 6B, C). Thus, in CTCs derived from heavily treated patients from advanced breast cancer, elacestrant can modulate ER activity at concentrations below those required for full protein degradation.

## Discussion

The recent development of potent oral SERDS, such as elacestrant, has the potential to provide a novel therapeutic intervention for patients with metastatic HR+ breast cancer, in whom the current standard of care, fulvestrant, has only modest benefit and it requires inconvenient intramuscular administration. Moreover, from a biological standpoint, the application of a highly effective degrader of ER provides important insight into the persistent dependency on this pathway by HR+ breast cancers that have become refractory to multiple currently available endocrine therapies. HR+ breast cancers that have progressed on drug combinations including AIs and SERMs may fail to respond to fulvestrant given the drug’s modest efficacy. However, a potent inhibitor such as elacestrant may readily distinguish advanced breast cancers with continued dependence on residual ER signaling, from those that have acquired other oncogenic drivers of malignancy. It is in this context that analysis of patient-derived CTC cultures, established from patients who have acquired resistance to multiple endocrine regimens, is particularly informative.

The CTC cultures analyzed here recapitulate the clinical observation that acquired *ESR1* mutations do not alter sensitivity to elacestrant [29]. Given the frequency of acquired *ESR1* mutations, particularly in patients who have had prolonged treatment with AI-containing combinations, and the dearth of other therapeutic options in such patients, these observations may be of significant clinical impact. ER degradation by fulvestrant and elacestrant has been demonstrated in multiple HR+ breast cancer cell lines (MCF7, T47D and HCC1428) that have not been exposed to therapeutic intervention [22]. Interestingly, in studying cultured breast CTCs from heavily treated patients, we find that the dose of elacestrant required to suppress ER transcriptional activity is lower than that required for degradation of ER protein, as measured by Western blotting. This raises the intriguing possibility that the cellular machinery required for enhanced ER degradation by elacestrant may be compromised in some advanced breast cancer cells, with elacestrant binding leading to ER modulation rather than degradation at lower drug concentrations.

The most striking observation reported here is that, despite both fulvestrant and elacestrant being classified as SERDS, previous treatment with fulvestrant does not affect the likelihood of response to elacestrant in cultured breast CTCs. This finding is consistent with our subgroup reanalysis of the EMERALD randomized trial. While the mechanisms underlying this lack of overlap in drug resistance are uncertain, it is possible that the modest effect by fulvestrant on ER degradation allows for adaptation by cancer cells to lower levels of ER activity; such cells may still be dependent on low levels of ER activity and hence susceptible to its complete abrogation by elacestrant, both in vitro and in vivo. In this regard, the unusual transient and reversible fulvestrant-resistant phenotype is striking in comparison with the prolonged proliferation arrest induced by elacestrant, a difference that may again reflect different degrees of suppression of ER activity. From the clinical standpoint, understanding patterns of acquired drug resistance may help guide the sequencing of endocrine therapies. For instance, a recent analysis of the EMERALD clinical trials data shows that a longer duration of prior CDK4/6 inhibitor treatment is associated with a longer PFS on elacestrant, as compared with SOC [35].

Of various next generation SERDS recently tested in randomized phase 3 clinical trials, elacestrant (pivotal phase 3) and camizestrant (phase 2) met its designated endpoint and achieved an improvement in PFS, while clinical trials evaluating other SERDs (amcenestrant, giredestrant) did not meet their primary endpoints [21, 29]. Further study will be required to determine if this difference relates to distinct intrinsic properties of the drugs themselves and their effect on ER activity, or on the differential composition of the study population, including the relatively higher fraction of *ESR1*-mutant cases in the EMERALD study [48%, compared with 43% (AMEERA-3), 39% (AcelERA), and 37% (SERENA-2)] [35–38]. In this context, the treatment-associated acquisition of *ESR1* mutations, which presents a major therapeutic challenge with current approved hormonal agents, may in fact serve to predict continued cancer dependence on ER activity and hence susceptibility to FDA-approved elacestrant, independent of prior fulvestrant therapy.

## Supplementary Information

Below is the link to the electronic supplementary material.

Supplementary file1 (PDF 449 KB)—Fig. 1 Treatment histories of metastatic breast cancer patients harboring mutant ESR1 (**A**) or wild type *ESR1* (**B**). Red arrow indicates the timepoint at which CTCs were collected and cultures were generated. Blue arrows indicate time of diagnosis of metastatic cancer and patient death. Fig. 2 *ESR1*-mutant CTCs with fulvestrant resistance are sensitive to elacestrant. *ESR1* mutant CTC lines Brx68 (**A**) and Brx211 (**B**) were treated with fulvestrant (100nM) for 7 days. The resistant cells were then re-treated with fulvestrant or with elacestrant for 7 days. Graphs show relative cell viability. Fig. 3 *ESR1*-wildype CTCs with fulvestrant resistance are sensitive to elacestrant. The *ESR1* wildtype CTC line Brx390 was treated with fulvestrant (100nM) for 7 days. The resistant cells were then re-treated with either fulvestrant or with elacestrant for 7days. Graphs show relative cell viability. Fig. 4 Long term treatment of elacestrant displays a persistent phenotype The effect of elacestrant on ER protein levels is independent of changes in *ESR1* mRNA expression. MCF7 cells were treated with increasing concentrations of elacestrant (DMSO, 0.5, 5 and 500 nM) for various times and *ESR1* mRNA expression was measured using qPCR.

## Data Availability

Additional data are available on request from the authors.
