# Supplementary material for: Modeling the novel SERD elacestrant in cultured fulvestrant-refractory HR-positive breast circulating tumor cells
Source: Breast Cancer Res Treat. 2023 Jun 15;201(1):43–56. doi: 10.1007/s10549-023-06998-w (PMC10300156; doi:10.1007/s10549-023-06998-w)

Supplementary Table 1

Mutational Profile for mutant *ESR1* patient-derived CTC lines

**BRx50 CTC Line****		
**Gene Mutation**	**Protein Change**	**AF**
ADAMTSL3	Arg185Pro	0.49
BRCA2¶	Leu2039fs	–
CIITA	Ala5Thr	0.52
ESR1	Leu536Pro	0.06††
FANCG	Asn167Ser	0.47
FEV	Trp87Ter	0.52
FPGT-TNNI3K,TNNI3K	Ala238Thr	0.47
GPC3	Arg203Cys	0.03
IGF2R	Arg1580Ser	0.99
IKZF1	Gly482Cys	0.09
IRAK4	Thr9Lys	0.38
JAK3	Arg403Cys	0.44
KLHL4	Pro419Ala	0.46
LRP1B	Lys1160Arg	0.48
NCOA1	Pro388His	0.52
NOTCH2	Pro1421Thr	0.5
NUP98	Ser1786Arg	0.47
PLAG1	Ala376Ser	0.48
TIAM1	Arg23Cys	0.5

**BRx68 CTC Line****		
**Gene Mutation**	**Protein Change**	**AF**
BCL9	Ser728Cys	0.02
CHD1L	Ser889Cys	0.14
CSNK1A1L	Glu41Lys	0.99
DCLK1	Tyr564Tyr	0.99
ESR1	Tyr537Ser	0.47
MSN	Glu385Lys	0.25
NTRK1	Ile7Thr	0.3
NUTM1	Leu684Leu	0.23
P2RY8	Tyr104Tyr	0.24
PIK3CA	His1047Arg	0.7
TIAM1	Pro1467Leu	0.51
TP53	Arg337Cys	0.99
TTN	Arg1436Ile	0.01

**Brx211 CTC Line#**		
**Gene Mutation**	**Protein Change**	**AF**
AURKA	Splice site	0.546
CDH1	Ile650TyrfsTer13	0.956
ESR1	Asp538Gly	1
PIK3CA	Glu110del	0.949
TSC2	Arg1369Trp	0.33

¶ Germline BRCA2 mutation was detected as part of genetic counseling for familial breast cancer.
– Insufficient Tumor tissue.

Ter Chain terminating codon.

†† ESR1 T1607C mutant allele frequency increased to 0.49 after prolonged *in vitro* culture under low-estrogen conditions (>6 months).

** Adapted from Yu et al. Science 2014

Adapted from Brett et al. JCO Precision Oncology 2023

Supplementary Table 2

Mutational Profile for wild type *ESR1* patient-derived CTC lines

Brx250 CTC Line#		
Gene Mutation	Protein Change	AF
ATM	Asp1853Val	0.526
ATM	Lys2317Asn	0.367
ATM	Glu1267Lys	0.503
ATRX	Ser342Phe	0.336
ATRX	Ser576Ter	0.447
CDH1	Asn405ArgfsTer9	0.924
ERBB3	Pro30Leu	0.504
ERBB4	Splice site	0.367
KRAS	Gln61His	0.378
MET	Thr1010Ile	0.505
NF1	Ile86Leu	0.071
NKX2-1	Ser79Trp	0.38
TP53	Glu285Lys	0.994

Brx390 CTC Line		
Gene Mutation	Protein Change	AF
CDH1	Splice site	0.971
CIC	Asn1283Asp	0.513
SMARCA4	Arg1639His	0.461
TP53	Gln192Ter	0.171

Brx394 CTC Line#		
Gene Mutation	Protein Change	AF
PIK3CA	Glu542Lys	0.465
RB1	Gln597His	0.976

Ter Chain terminating codon.

Adapted from Brett et al. JCO Precision Oncology 2023

Supplementary Figure 1A

Treatment History of patients: mutant *ESR1* CTC lines

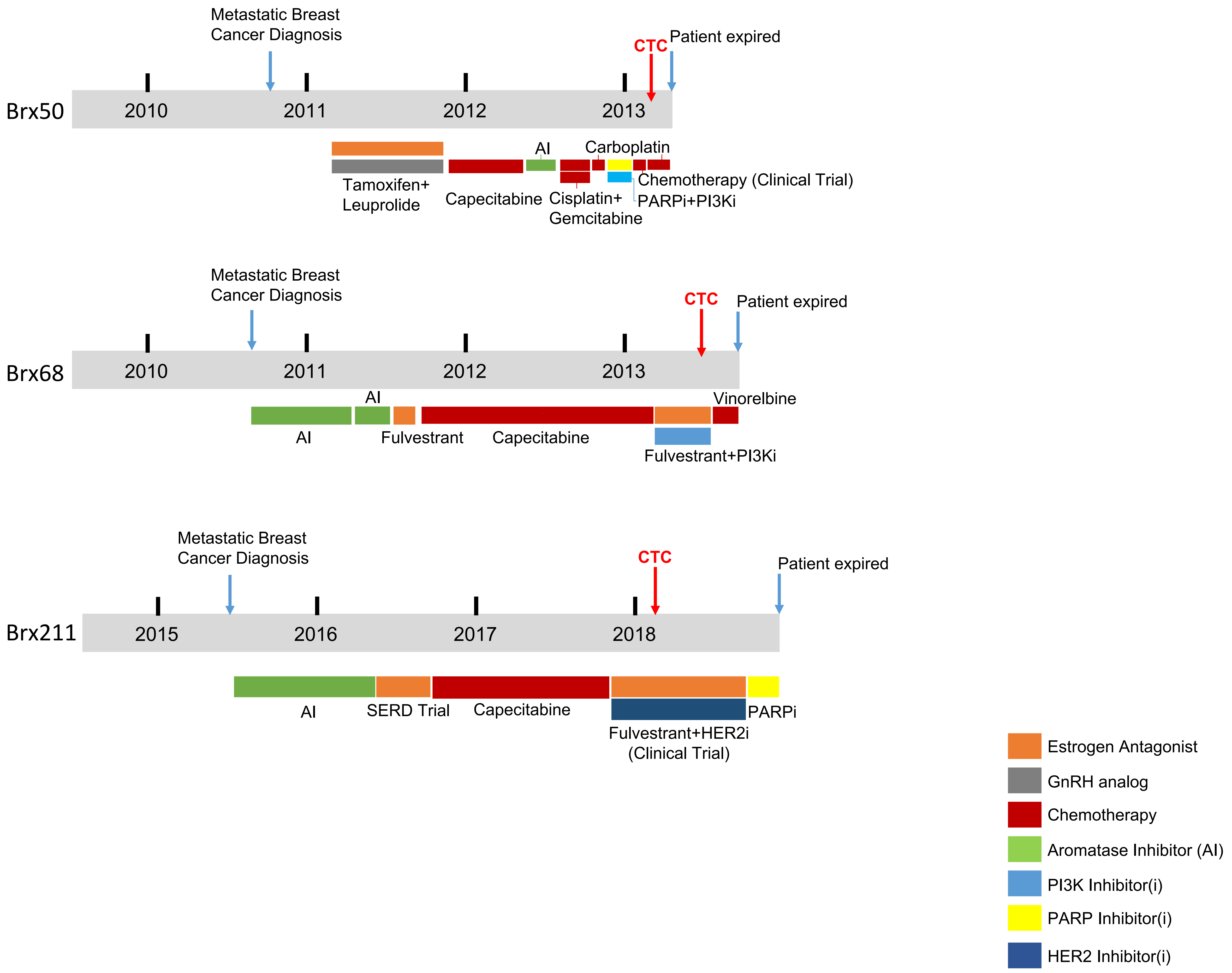

Supplementary Figure 1B

Treatment History of patients: wild type *ESR1* CTC lines

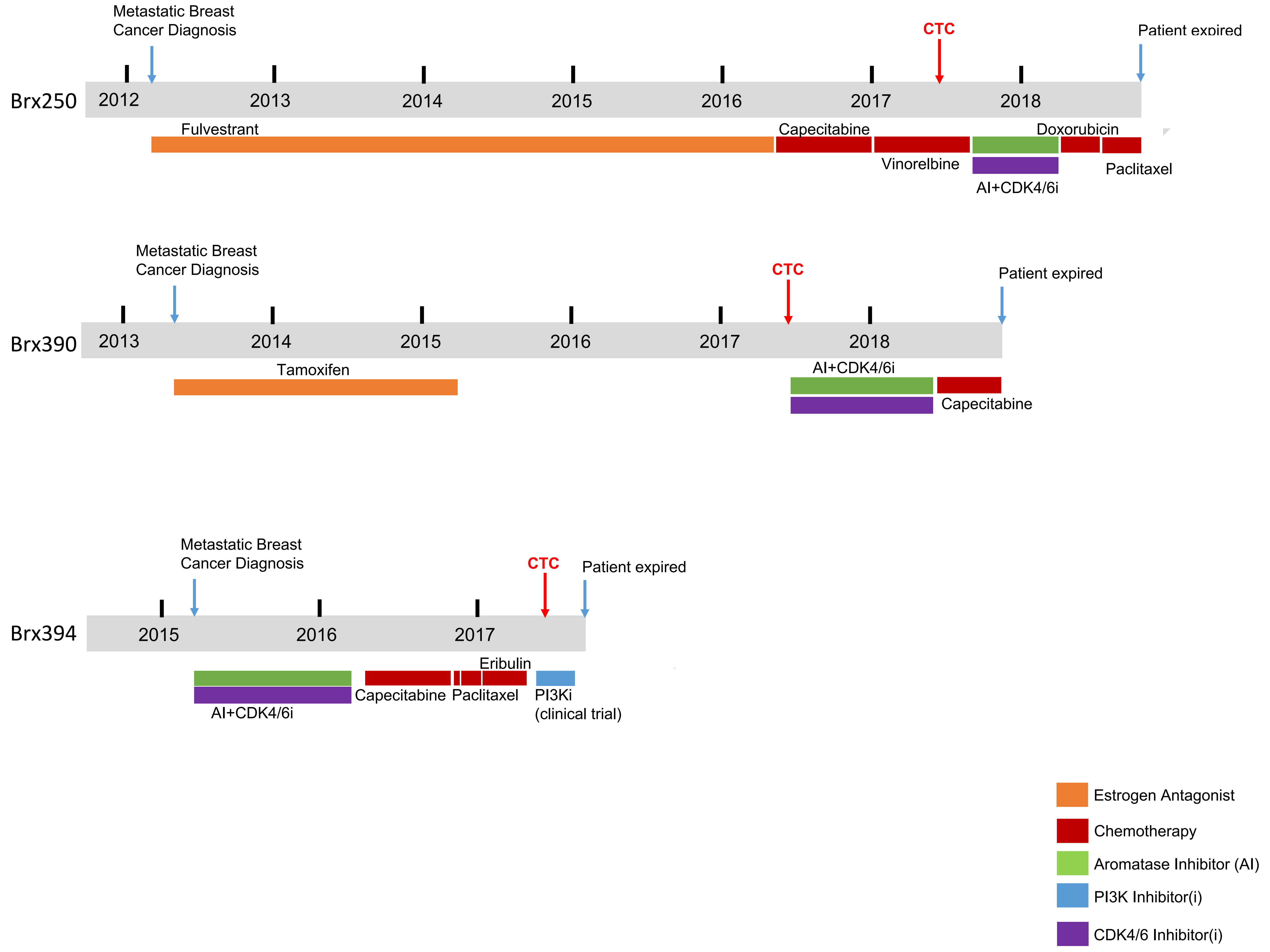

Supplementary Figure 2

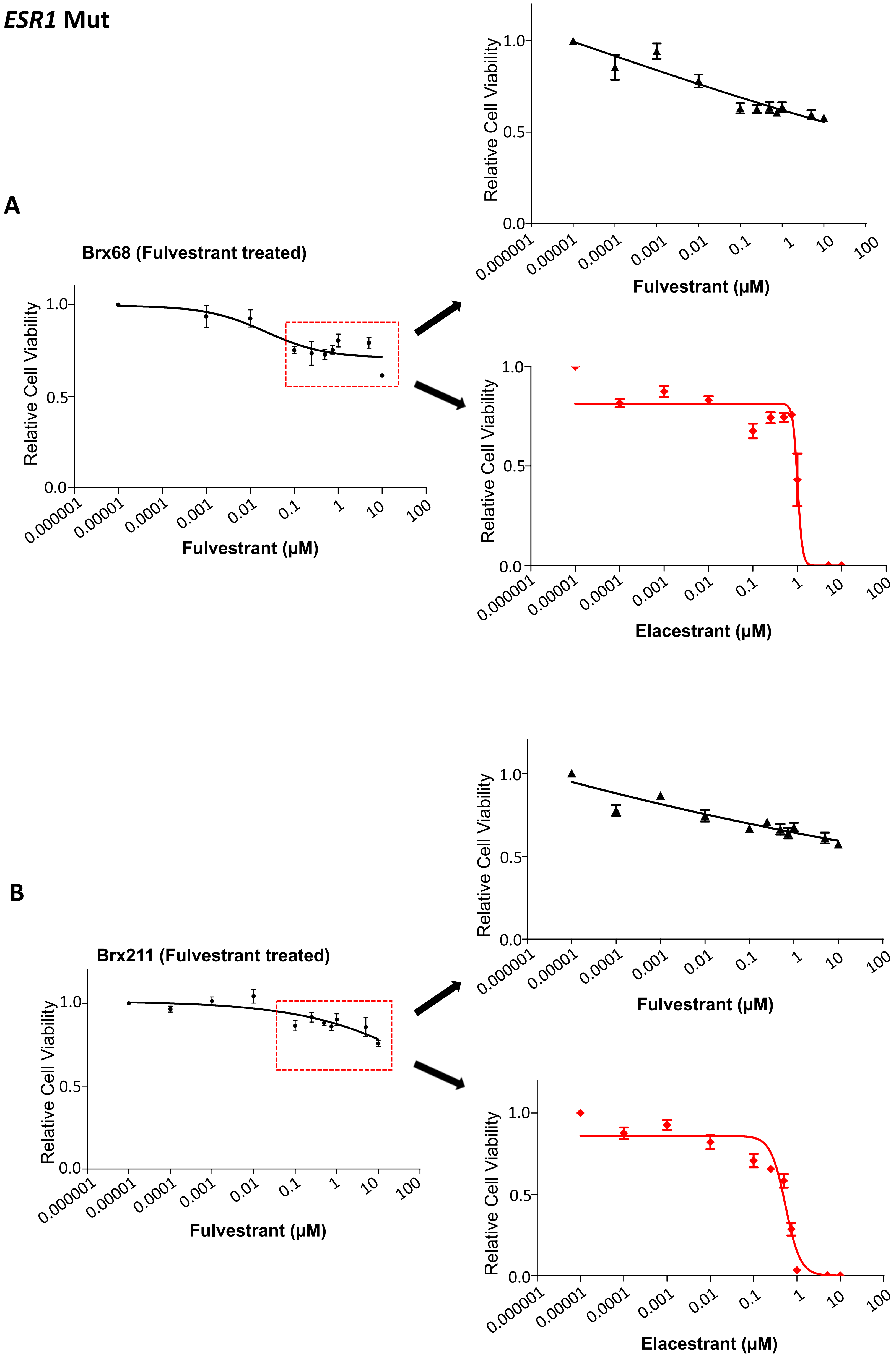

Supplementary Figure 3

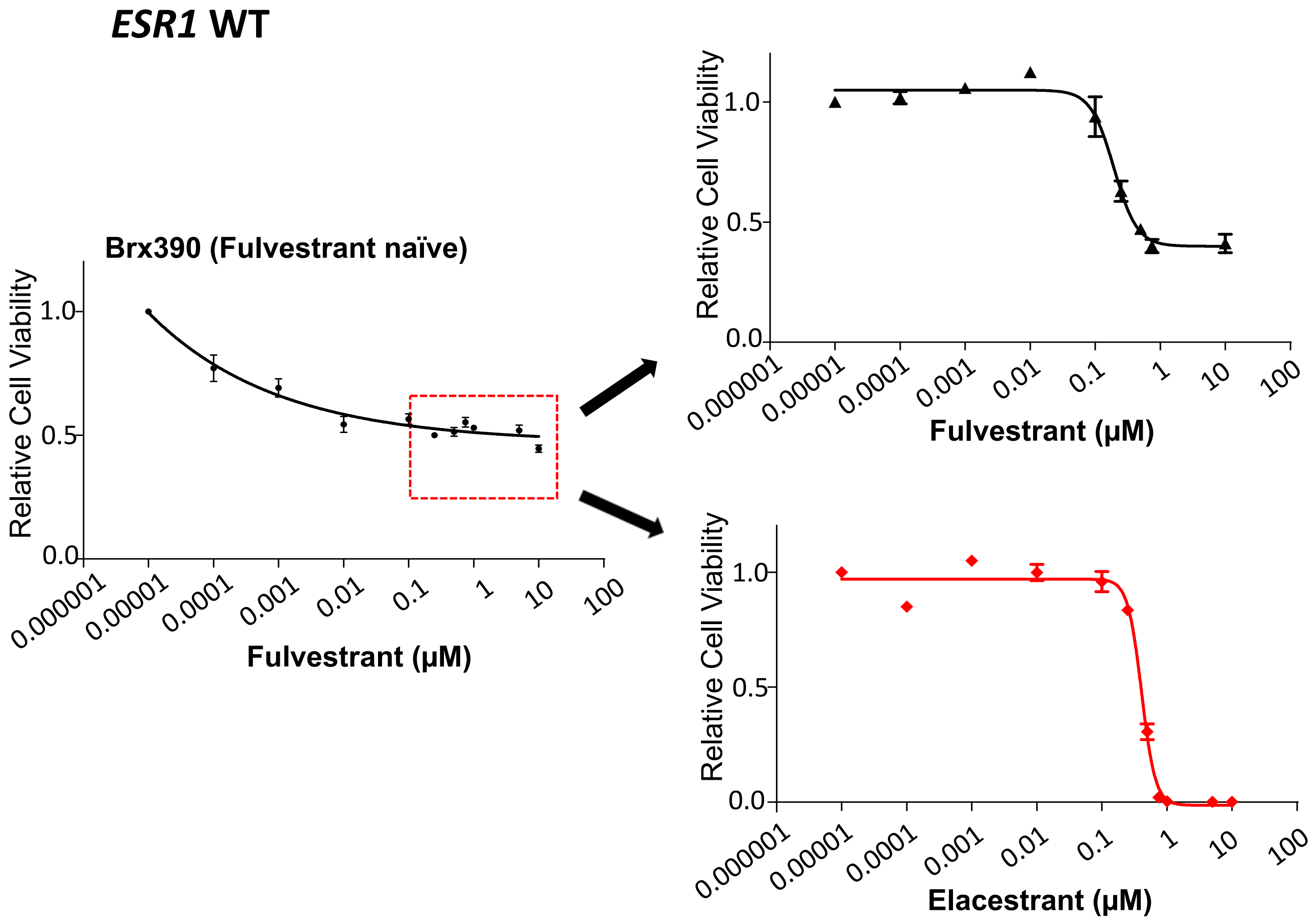

Supplementary Figure 4

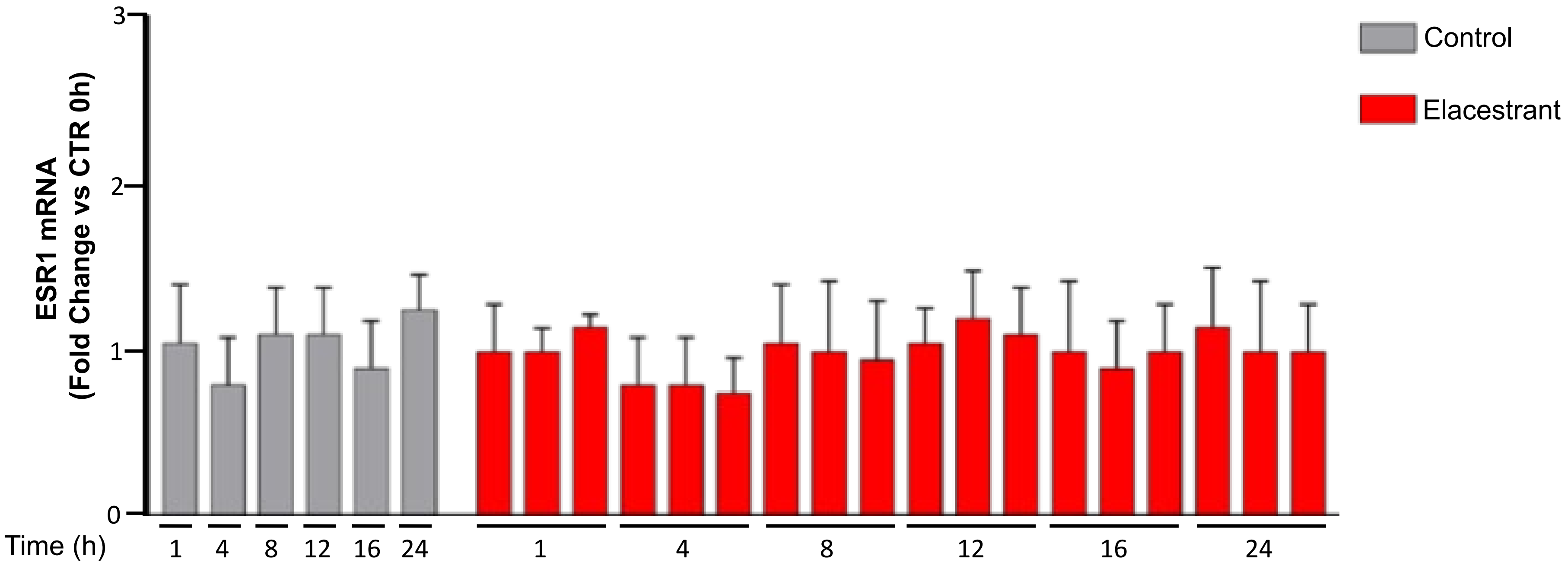

Supplement: Supplementary file 1 — Supplementary file1 (PDF 449 KB)—Fig. 1 Treatment histories of metastatic breast cancer patients harboring mutant ESR1 (A) or wild type ESR1 (B). Red arrow indicates the timepoint at which CTCs were collected and cultures were generated. Blue arrows indicate time of diagnosis of metastatic cancer and patient death. Fig. 2 ESR1-mutant CTCs with fulvestrant resistance are sensitive to elacestrant. ESR1 mutant CTC lines Brx68 (A) and Brx211 (B) were treated with fulvestrant (100nM) for 7 days. The resistant cells were then re-treated with fulvestrant or with elacestrant for 7 days. Graphs show relative cell viability. Fig. 3 ESR1-wildype CTCs with fulvestrant resistance are sensitive to elacestrant. The ESR1 wildtype CTC line Brx390 was treated with fulvestrant (100nM) for 7 days. The resistant cells were then re-treated with either fulvestrant or with elacestrant for 7days. Graphs show relative cell viability. Fig. 4 Long term treatment of elacestrant displays a persistent phenotype The effect of elacestrant on ER protein levels is independent of changes in ESR1 mRNA expression. MCF7 cells were treated with increasing concentrations of elacestrant (DMSO, 0.5, 5 and 500 nM) for various times and ESR1 mRNA expression was measured using qPCR. [file 10549_2023_6998_MOESM1_ESM.pdf]
